# Supplementary material for: Substrate fluxes in brown adipocytes upon adrenergic stimulation and uncoupling protein 1 ablation
Source: Life Sci Alliance. 2018 Nov 14;1(6):e201800136. doi: 10.26508/lsa.201800136 (PMC6238590; doi:10.26508/lsa.201800136)
Supplement: Supplementary file 1 [file LSA-2018-00136_TableS1.docx]

|  | **fold change** | **p** |
| --- | --- | --- |
| Irf4 | 2,3 | 0,035 |
| Grem1 | -4,0 | 0,000 |
| Vtn | -3,6 | 0,017 |
| Kng1 | -3,5 | 0,020 |
| Comp | -3,5 | 0,031 |
| Ucp1 | -3,3 | 0,000 |
| Hoxb9 | -3,3 | 0,025 |
| Cfi | -3,0 | 0,021 |
| Cfh | -3,0 | 0,024 |
| Galnt9 | -3,0 | 0,021 |
| Gdf10 | -3,0 | 0,011 |
| Igfbp2 | -2,8 | 0,004 |
| Lum | -2,7 | 0,043 |
| Sfrp1 | -2,7 | 0,003 |
| Cd200 | -2,7 | 0,025 |
| Clu | -2,6 | 0,022 |
| Rtn4rl2 | -2,2 | 0,026 |
| Arhgap20 | -2,2 | 0,040 |
| Gcnt4 | -2,1 | 0,045 |
| 2900026A02Rik | -2,1 | 0,004 |
